# Prognostic value of *TMTC1* in pan-cancer analysis

**DOI:** 10.1016/j.heliyon.2024.e38308

**Published:** 2024-09-27

**Authors:** Ying Zhang, Dan Wu, Tiantian Yu, Yao Liu, Chunbo Zhao, Ruihong Xue

**Affiliations:** a The International Peace Maternity and Child Health Hospital, School of Medicine, Shanghai Jiao Tong University, Shanghai, China; b Shanghai Key Labouratory of Embryo Original Diseases, 200030, Shanghai, China; c Institute of Birth Defects and Rare Diseases, School of Medicine, Shanghai Jiao Tong University, 200030, Shanghai, China; d Department of Obstrics and Gynecology, The First People's Hospital of Jiande, Hangzhou, China

**Keywords:** *TMTC1*, Pan-cancer, Prognosis, Immunotherapy

## Abstract

**Background:**

Transmembrane and tetratricopeptide repeat containing 1 (*TMTC1*) is a recently discovered enzyme involved in the O-mannosylation of cadherins and protocadherins. It has been implicated in various types of cancer, but the overall prognostic significance of *TMTC1* in pan-cancer and its potential as an immunotherapeutic target remain unclear.

**Methods:**

We applied various bioinformatics methods to investigate the potential oncogenic roles of *TMTC1* using public databases. This analysis involved examining the expression, prognosis, genetic alterations, immune infiltration, immunotherapy response, drug sensitivity, and regulatory mechanisms of the *TMTC1* gene in diverse cancer types.

**Results:**

In this study, we observed that *TMTC1* expression is reduced in 19 types of cancer (ACC, BLCA, BRCA, CESC, COAD, ESCA, GBM, KICH, KIRC, KIRP, LAML, LUAD, LUSC, PRAD, READ, STAD, THCA, UCEC, and UCS) compared to normal tissues. Conversely, *TMTC1* expression is elevated in OV and PAAD relative to normal tissues. Moreover, our analysis revealed that high expression of *TMTC1* was associated with worse overall survival (OS) outcomes in patients with ACC, BLCA, COAD, GBM, KIRP, OV, STAD, and UCEC, but better OS outcomes in patients with CESC, KIRC, LUSC, and PAAD. Notably, patients with *TMTC1* mutations or deep deletions demonstrated longer OS, while those with *TMTC1* amplification showed shorter OS. There was a significant correlation between the expression level of *TMTC1* and the infiltration of cancer-associated fibroblasts (CAFs) and endothelial cells. Using data from six real-world immunotherapy cohorts of BLCA, SKCM and RCC, we discovered that high *TMTC1* expression was associated with better OS or progression-free survival (PFS). Lastly, through *TMTC1*-related gene enrichment analysis, some biological processes and pathways were found to be significantly enriched, such as vascular endothelial growth factor receptor signaling pathway and ECM-receptor interaction.

**Conclusions:**

Our study demonstrates the prognostic significance of *TMTC1* in pan-cancer and highlights its potential as an immunotherapeutic target.

## 1 Introduction

Cancer, characterized by the abnormal growth and spread of cells, remains a major global health concern, causing substantial morbidity and mortality [1,2]. To develop effective therapeutic strategies, it is imperative to gain a deep understanding of the intricate molecular mechanisms responsible for the initiation and progression of cancer. Recent years have witnessed remarkable advancements in high-throughput sequencing technologies, revolutionizing the exploration of molecular alterations across different types of cancer. Consequently, the identification and characterization of cancer-related genes and their involvement in tumor formation and development have emerged as indispensable undertakings in the field of cancer research.

The TMTC (transmembrane (TM) and tetratricopeptide (TPR) repeat-containing) proteins family consists of four members in humans, namely *TMTC1*-4, and serves as resident proteins of the endoplasmic reticulum (ER) [3]. The TMTCs proteins have an N-terminal composed of 11 transmembrane regions and multiple intermittent loops, while their C-terminal is located in the ER lumen and consists of TPR repeats [[3], [4], [5]]. The TMTCs proteins play essential roles in ER function. For example, *TMTC1* and TMTC2 can interact with the ER calcium uptake pump SERCA2B via their C-terminal TPR domains, participating in the regulation of ER calcium homeostasis [5]. TMTC3 interacts with PDIA3 and regulates proteasome activity and the expression of XBP-1, a stress response protein, thereby participating in the ER stress response [4]. Similarly, TMTC4 also interacts with the ER calcium pump SERCA2b, altering ER calcium dynamics, which leads to overactivation of the downstream unfolded protein response and cell death [6]. Recent studies have revealed that TMTCs function as glycosyltransferases, playing a crucial role in the O-mannosylation process of cadherins [7] and integrin β [8]. Consequently, they actively contribute to the progression of cancer [3,7,9,10].

*TMTC1*, as an important member of the TMTC family, plays a crucial role in various physiological pathways. Studies have revealed its involvement in protein glycosylation [3], endoplasmic reticulum calcium homeostasis [5], cellular adhesion [8], cell differentiation [11], and inflammation [12]. Furthermore, accumulating evidence suggests that dysregulation of *TMTC1* is associated with several human diseases, including cancer. For instance, *TMTC1* mutations have been linked to schizophrenia [13]. In the context of cancer, *TMTC1* is upregulated in ovarian cancer and promotes migration and invasion of ovarian cancer cells through modifications in O-mannosylation and the activity of integrins β1 and β4 [8]. High *TMTC1* mRNA expression is also correlated with decreased survival in gastric cancer patients [14]. Additionally, *TMTC1* has been identified as being involved in multiple other cancers, such as breast cancer [15,16]、renal cancer [16]、glioblastoma [17] and nasopharyngeal carcinoma [18]. Although previous studies have suggested the potential role of *TMTC1* in cancer, a comprehensive analysis of its expression and prognostic significance across different types of cancer is currently lacking. Hence, it is necessary to systematically examine *TMTC1* expression in multiple cancer types in order to unravel its involvement in carcinogenesis and evaluate its potential as a prognostic biomarker.

Our research is the first pan-cancer analysis of *TMTC1* by utilizing TCGA, GTEx, HPA, and GEO databases. Our analysis comprehensively examined several critical aspects including gene expression, prognosis, genetic alterations, immune infiltration, immunotherapy response, drug sensitivity, and regulatory mechanisms of *TMTC1* in pan-cancer. By elucidating the role of *TMTC1* in different cancer types, this study may pave the way for targeted therapeutic interventions that could improve patient outcomes and potentially provide novel avenues for cancer treatment.

## 2 Materials and methods

### 2.1 Data acquisition

Gene expression data, clinical phenotype data, and genetic alterations (mutations and copy number variations) data of thirty-three cancer types form TCGA were acquired from the UCSC Xena database (https://xenabrowser.net/datapages/) [19]. The thirty-three cancers of interest in this study, with their full names and abbreviations, are presented in Table 1. Gene expression profile of human normal tissues were retrieved from GTEx (https://commonfund.nih.gov/GTEx) [20]. Two immunotherapy cohorts of SKCM were obtained from the European Nucleotide Archive (https://www.ebi.ac.uk/ena/browser/view/PRJEB23709) and GEO (https://www.ncbi.nlm.nih.gov/geo/query/acc.cgi?acc=GSE91061), respectively [21,22]. Two immunotherapy cohorts of BLCA [23,24] were obtained from available data package (http://research-pub.gene.com/IMvigor210CoreBiologies) and GEO (https://www.ncbi.nlm.nih.gov/geo/query/acc.cgi?acc=GSE176307). Two immunotherapy cohorts of RCC were obtained from available data package (http://research-pub.gene.com/IMvigor210CoreBiologies) and Motzer's articles [25]. An immunotherapy cohort of KIRC cohort were extracted from the supplementary files of Braun's articles [26]. The IC50 values and transcriptomic data for cell lines treated with 198 drugs were downloaded from the GDSC2 database (https://www.cancerrxgene.org).

**Table 1:** List of cancer types.

Study Abbreviation	Study Name
ACC	Adrenocortical carcinoma
BLCA	Bladder Urothelial Carcinoma
BRCA	Breast invasive carcinoma
CESC	Cervical squamous cell carcinoma and endocervical adenocarcinoma
CHOL	Cholangiocarcinoma
COAD	Colon adenocarcinoma
DLBC	Lymphoid Neoplasm Diffuse Large B-cell Lymphoma
ESCA	Esophageal carcinoma
GBM	Glioblastoma multiforme
HNSC	Head and Neck squamous cell carcinoma
KICH	Kidney Chromophobe
KIRC	Kidney renal clear cell carcinoma
KIRP	Kidney renal papillary cell carcinoma
LAML	Acute Myeloid Leukemia
LGG	Brain Lower Grade Glioma
LIHC	Liver hepatocellular carcinoma
LUAD	Lung adenocarcinoma
LUSC	Lung squamous cell carcinoma
MESO	Mesothelioma
OV	Ovarian serous cystadenocarcinoma
PAAD	Pancreatic adenocarcinoma
PCPG	Pheochromocytoma and Paraganglioma
PRAD	Prostate adenocarcinoma
RCC	Renal cell carcinoma
READ	Rectum adenocarcinoma
SARC	Sarcoma
SKCM	Skin Cutaneous Melanoma
STAD	Stomach adenocarcinoma
TGCT	Testicular Germ Cell Tumors
THCA	Thyroid carcinoma
THYM	Thymoma
UCEC	Uterine Corpus Endometrial Carcinoma
UCS	Uterine Carcinosarcoma
UVM	Uveal Melanoma

### 2.2 Expression analysis

The expression data of the *TMTC1* gene (ENSG00000133687) were extracted from downloaded transcriptome data. Following TPM normalization, we conducted a log2(x+1) conversion. In order to enhance reliability and control for possible batch effects, data from the same sample, but acquired from different test batches, were averaged. Furthermore, we filtered the samples from the following sources: Solid Tissue Normal, Primary Solid Tumor, Primary Tumor, Normal Tissue, Primary Blood Derived Cancer - Bone Marrow, and Primary Blood Derived Cancer - Peripheral Blood. We also excluded cancer types with less than 3 samples, resulting in a final dataset of expression data from 28 cancer and their corresponding adjacent normal tissues. We used R software (version 4.0.2) to calculate the expression differences of *TMTC1* between tumor and normal samples in each tumor type. Additionally, we assessed the expression differences of *TMTC1* in different clinical stage samples using Wilcoxon's test for significance analysis. The results were visualized using the “ggpubr” R package [27].

The Human Protein Atlas (HPA) database [28] (https://www.proteinatlas.org/) is a comprehensive database of human protein information, aiming to provide a detailed description of human gene and protein expression patterns. This database integrates proteomic data from various tissue and cell types, including immunohistochemistry, immunofluorescence, and high-throughput antibody preparation techniques. Utilizing this database, we obtained immunohistochemical data of *TMTC1* protein in distinct cancerous and normal tissues, enabling us to examine the differential protein expression profiles at a protein level. Furthermore, to elucidate the cellular localization of the *TMTC1* protein, we also obtained immunofluorescence data from U-251-MG cell line treated with *TMTC1* antibodies from this database.

### 2.3 Survival analysis

The “survival” R package [29] was employed to conduct survival analysis on the patient data. To establish an optimal cutoff point for continuous *TMTC1* expression, the surv_cutpoint function was utilized. Subsequently, an analysis was performed to correlate the expression levels of *TMTC1* with OS. To visualize the results, forest plots and Kaplan-Meier (KM) curves plots were generated using the “forestplot” [30] and “survminer” [31] R packages, respectively.

### 2.4 Genetic alteration analysis

In order to explore the specific characteristics of genetic alterations in *TMTC1* across various cancers. we conducted a comprehensive study on the incidence and number of mutations, amplifications, and deep deletions of *TMTC1* in 32 different types of cancers. Furthermore, the impact of *TMTC1* genetic alterations on patient survival was assessed using the "survival" R package. Survival analyses comparing patients with *TMTC1* mutations, amplifications, and deep deletions to those without any alterations were conducted, and the results were visualized using Kaplan-Meier (KM) curve plots.

### 2.5 Immune infiltration analysis

To investigate the relationship between *TMTC1* expression and immune infiltration, we initially employed the "Immune Infiltration" module of the SangerBox3.0 [32] platform. This module allowed us to compute the Pearsons's correlation coefficient between *TMTC1* expression and immune infiltration scores, including stromal, immune, and ESTIMATE scores [33], across various tumor samples, resulting in the identification of significant correlations. The correlation analysis results were then visualized as a heatmap using the "pheatmap" [34] R package.

To explore the relationship between *TMTC1* expression and the abundance of cancer-associated fibroblasts and endothelial cells in the tumor microenvironment, we utilized the "Gene" module in TIMER2.0 (http://timer.cistrome.org/), which provides several immune deconvolution methods for calculating the abundance of cancer-associated fibroblasts and endothelial cells including EPIC [35], MCP-counter [36], XCELL [37], and TIDE. The Spearman's correlations were calculated and a heatmap with numbers showed the purity-adjusted spearman's rho across various cancer types. By clicking on specific cells within the heatmap, scatter plots illustrating the relationship between infiltrate estimation values and gene expression in different types of cancer were generated.

### 2.6 Single-cell analysis of TMTC1

To investigate the correlation between *TMTC1* and the tumor microenvironment at the single-cell level, we utilized the "Gene Exploration" module in the Tumor Immune Single-cell Hub (TISCH) database (http://tisch.comp-genomics.org/home/) [38]. This module enabled quantification of *TMTC1* expression in immune cells and stromal cells within the tumor microenvironment. The following main parameters were set: gene selected as “*TMTC1*”, cell-type annotation selected as “Celltype (maior-lineage)”, cancer type selected as “BLCA, BRCA, CHOL, KIRC, LIHC, UVM, UCEC, STAD, SKCM, SARC, PAAD, OV, and HHSC”. To explore the expression of *TMTC1* in single-cell data of a specific cancer, the "Dataset" module provided the option to click on a specific dataset name, followed by inputting "*TMTC1*″ in the "Gene" module, resulting in the generation of heatmaps and violin plots displaying *TMTC1* expression.

### 2.7 Immunotherapy outcome analysis

Immunotherapy has emerged as a promising approach for treating various diseases, including cancer [39]. To explore the potential prognostic value of *TMTC1* in the context of immunotherapy, we collected data from six immunotherapy cohorts of four types of cancer, along with their transcriptomic profiles and clinical information (see the data acquisition section above for more details). We then performed survival analysis on patients with different expression levels of *TMTC1* (see the survival analysis section for methods).

### 2.8 Drug sensitivity analysi**s**

The GDSC2 (Genomics of Drug Sensitivity in Cancer version 2) database (https://www.cancerrxgene.org) is a comprehensive database aimed at studying the sensitivity of cancer drugs [40]. This database integrates various data on cancer cell lines, including genomics, transcriptomics, and drug responses. To analyze the correlation between drug sensitivity and *TMTC1* expression, IC50 values and transcriptomic data for cell lines treated with 198 drugs were downloaded from the GDSC2 database. The “oncoPredict” [41] R package was used to calculate the IC50 for each sample from TCGA, using the transcriptomic data from GDSC2 as the training group and transcriptomic data from TCGA as the verification group. Pearson's correlation coefficient was computed to assess the relationship between drug IC50 values and *TMTC1* expression across pan-cancer samples. Heatmaps, scatter plots, and box plots were generated using the "pheatmap" [34] and "ggpubr" R packages for visualization.

### 2.9 TMTC1-related gene enrichment analysis

The LinkedOmics database (http://www.linkedomics.org/login.php) [42] was utilized to identify *TMTC1*-related genes in ovarian serous cystadenocarcinoma. The following main parameters were set: cancer type selected as ovarian serous cystadenocarcinoma, search dataset selected as "RNA-seq data type, HiSeq RNA platform", search dataset attribute narrowed down to *TMTC1*, target dataset specified as "RNA-seq data type, HiSeq RNA platform", and statistical method chosen as Pearson's correlation test. The top 100 genes, with 50 positively correlated and 50 negatively correlated with *TMTC1* in ovarian cancer, were displayed using a heatmap. The gene set enrichment analysis (GSEA) function module was applied to examine KEGG pathways and GO_BP terms, using a rank criterion of FDR <0.05 and performing 1000 simulations.

#### 2.9.1 Statistical analysis

Statistical analyses were performed using R 3.6.2 (https://www.r-project.org/). Differences between the two groups and among multiple groups were analyzed using the default Wilcoxon's test and one-way analysis of variance (ANOVA), respectively. The differences in overall survival between groups were determined by Kaplan-Meier analysis and a log-rank test. P value < 0.05 was considered to be statistically significant if not otherwise stated.

## 3 Results

### 3.1 Analysis of TMTC1 expression in pan-cancer

**Fig. 1:**
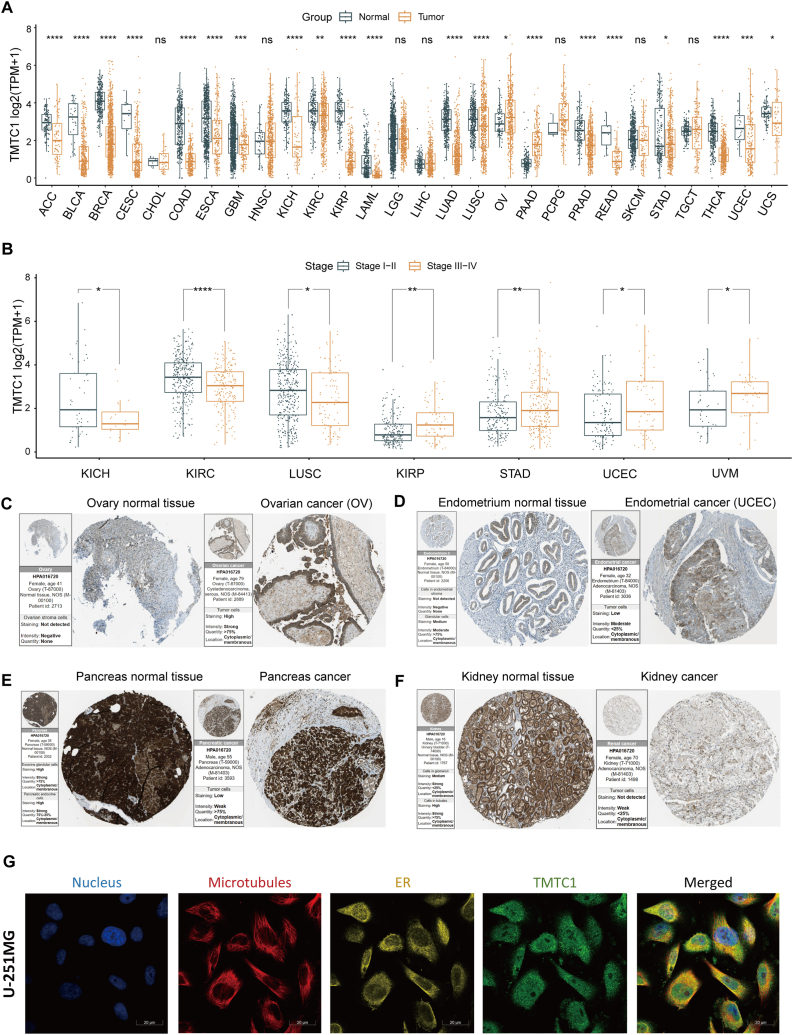
*TMTC1* expression analysis in pan-cancer. (A) *TMTC1* mRNA expression levels in the tumor and matched normal tissues of 28 organs. **(B)** The differential expression of *TMTC1* across different stages (stage I- II and stage III-IV) of 7 cancers. **(C**–**F)** The immunohistochemistry data of *TMTC1* in tumors and normal tissues of ovary **(C)**, endometrium **(D),** pancreas **(E)**, and kidney **(F)**. **(E)** The immunofluorescence data of *TMTC1* in U-251MG cell line from the human protein atlas. The antibody used was a rabbit polyclonal antibody (HPA016720) from Sigma-Aldrich. ∗p < 0.05; ∗∗p < 0.01; ∗∗∗p < 0.001, and ∗∗∗∗p < 0.0001. The statistical difference of two groups was compared through the Wilcox test.

To investigate the expression of *TMTC1* in different types of cancer, we firstly conducted a comprehensive study by comparing mRNA expression levels in cancer tissues and normal tissues using data from the TCGA and GTEx databases. As shown in Fig. 1A, noteworthy downregulation of *TMTC1* expression was observed in the majority of tumor tissues, including ACC, BLCA, BRCA, CESC, COAD, ESCA, GBM, KICH, KIRC, KIRP, LAML, LUAD, LUSC, PRAD, READ, STAD, THCA, UCEC, and UCS. Conversely, *TMTC1* was found to be upregulated in OV and PAAD. Additionally, we investigated whether *TMTC1* had an impact on cancer staging. As shown in Fig. 1B, our findings demonstrated significantly higher *TMTC1* expression in the early stages (Stage I and Stage II) of LUSC, KICR, and KIRC, while it was significantly higher in the late stages (Stage III and Stage IV) of KIRP, STAD, UCEC, and UVM. Moreover, analysis of *TMTC1* protein expression using immunohistochemistry data from the HPA database revealed a marked increase in OV (Fig. 1C) and UCEC (Fig. 1D) when compared to normal tissues. Conversely, *TMTC1* expression was significantly lower in pancreas (Fig. 1E) and kidney (Fig. 1F) cancer tissues compared to normal tissues, thus confirming the differential expression observed at the mRNA level. Finally, immunofluorescence data from the HPA revealed that *TMTC1* primarily was primarily located in the endoplasmic reticulum and microtubules.

### 3.2 Prognostic significance of TMTC1 expression in pan-cancer

**Fig. 2:**
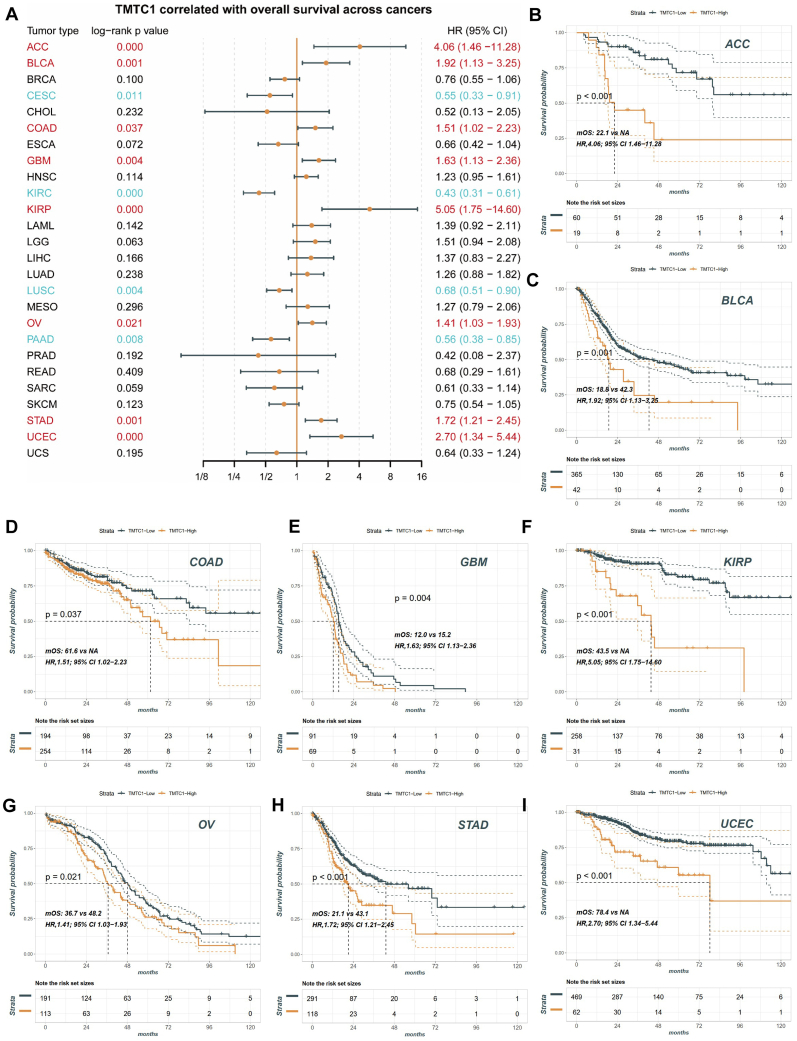
The prognostic significance of *TMTC1* expression in various types of cancer (A) The forest plots of univariate cox regression analyses for OS. The red bold (HR > 1) and blue bold (HR < 1) items indicated that *TMTC1* expression was significantly correlated with prognosis in these types of cancers (p < 0.05). **(B**–**I)** KM curves of overall survival analysis of *TMTC1* in ACC **(B)**, BLCA **(C)**, COAD **(D)**, GBM **(E)**, KIRP **(F)**, OV **(G)**, STAD **(H)** and UCEC **(I)**.

To investigate the potential prognostic value of *TMTC1* expression in pan-cancer patients, data from the TCGA database were analyzed. The patients were divided into two groups based on the level of *TMTC1* expression. Initially, we performed univariate Cox regression analysis to determine the association between *TMTC1* expression and survival. As shown in Fig. 2A, our findings revealed that *TMTC1* expression served as an independent risk factor for survival in patients with ACC (HR = 4.06, 95 % CI 1.46–11.28), BLCA (HR = 1.92, 95 % CI 1.13–3.25), COAD (HR = 1.51, 95 % CI 1.02–2.23), GBM (HR = 1.63, 95 % CI 1.13–2.36), KIRP (HR = 5.05, 95 % CI 1.75–14.60), OV (HR = 1.41, 95 % CI 1.03–1.93), STAD (HR = 1.72, 95 % CI 1.21–2.45), and UCEC (HR = 2.70, 95 % CI 1.34–5.44) (all log-rank p value < 0.05). Conversely, *TMTC1* expression appeared to have a protective effect on survival in patients with CESC (HR = 0.55, 95 % CI 0.33–0.91), KIRC (HR = 0.43, 95 % CI 0.31–0.61), LUSC (HR = 0.68, 95 % CI 0.51–0.90), and PAAD (HR = 0.56, 95 % CI 0.38–0.85) (all log-rank p value < 0.05). Moreover, Kaplan–Meier survival curves showed that *TMTC1* were associated with poorer OS in patients with ACC (Fig. 2B), BLCA (Fig. 2C), COAD (Fig. 2D), GBM (Fig. 2E), KIRP (Fig. 2F), OV (Fig. 2G), STAD (Fig. 2H), and UCEC (Fig. 2I).

### 3.3 Analysis of TMTC1 genetic alterations in pan-cancer

**Fig. 3:**
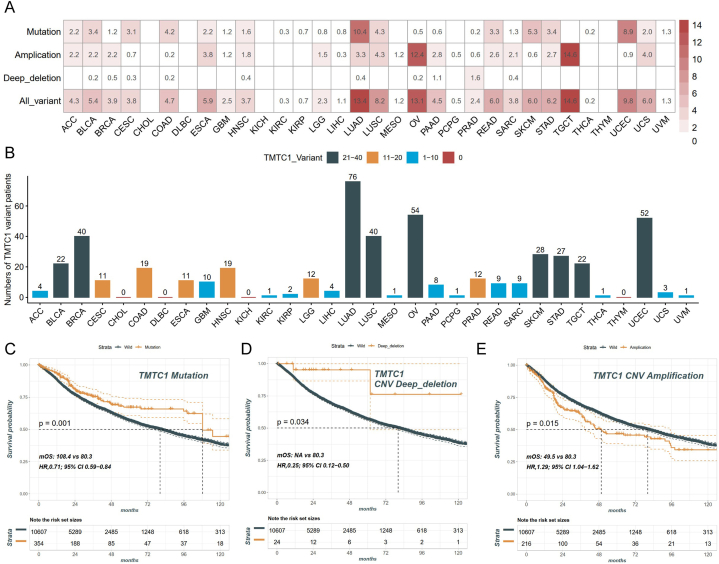
Genetic alteration characteristics of *TMTC1*. (A) The alteration (mutation, amplification, deep deletion, and all variants) frequency of *TMTC1* gene in 32 cancers from the TCGA dataset. (B) The number of patients with *TMTC1* alteration in 32 cancers from the TCGA dataset. (C-e) KM curves of overall survival analysis between patients with *TMTC1* mutation **(C)**, amplification **(D)**, deep deletion **(E)** and patients without any *TMTC1* alterations in pan-cancer.

It is widely recognized that gene mutations are closely associated with tumorigenesis**.** To examine the genetic variations in *TMTC1* across different types of cancers, we conducted a comprehensive analysis using genomic data obtained from the TCGA database across various types of cancer, As shown in Fig. 3A and B, among the 32 types of cancer patients, a total of 499 patients presented the *TMTC1* variant. Except for CHOL, DLBC, KICH, and THYM, all other 28 types of cancer patients showed the *TMTC1* variant. These variants occurred primarily in the form of mutation and amplification. The highest frequency of *TMTC1* variant was observed in LUAD, OV, and TGCT. Among these, LUAD had mutations as the primary alteration, while OV and TGCT presented amplification as the main change. Finally, our analysis examined the impact of *TMTC1* genetic alterations on the prognosis of cancer patients. The results revealed in Fig. 3A and B demonstrated that patients with *TMTC1* mutation and deep deletion had significantly better OS than the wild-type group. Conversely, patients with *TMTC1* amplification exhibited significantly worse OS compared to the wild-type group (Fig. 3C).

### 3.4 Immune infiltration analysis of TMTC1

**Fig. 4:**
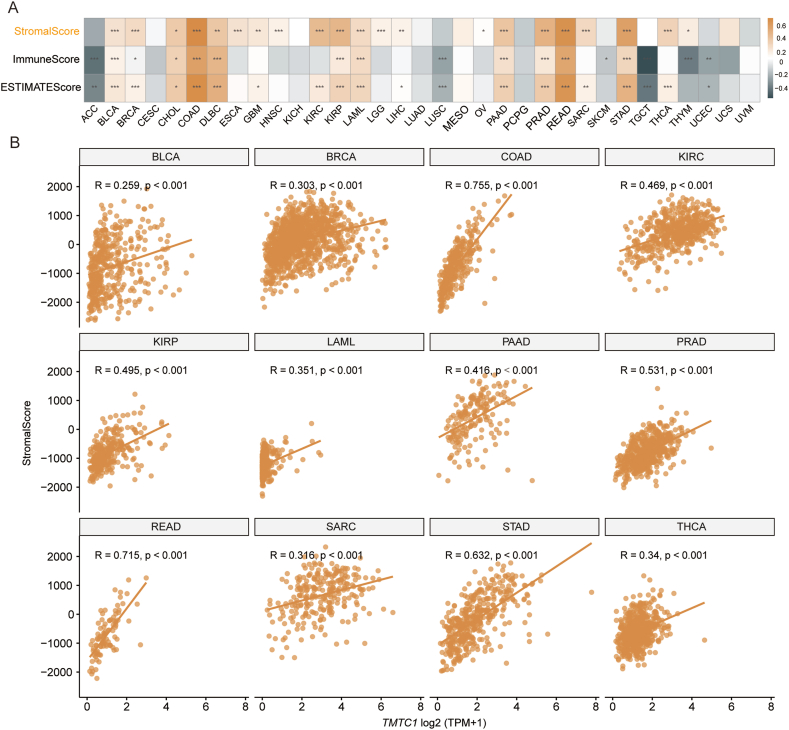
The association of *TMTC1* expression with ESTIMATEScore, ImmuneScore, and StromalScore in various types of cancer. (A) The heatmap showed the correlation between the expression of *TMTC1* in cancers and the ImmuneScore, StromalScore, and ESTIMATEScore. **(B)** The scatter plots of *TMTC1* expression and StromalScore in 12 cancers.

**Fig. 5:**
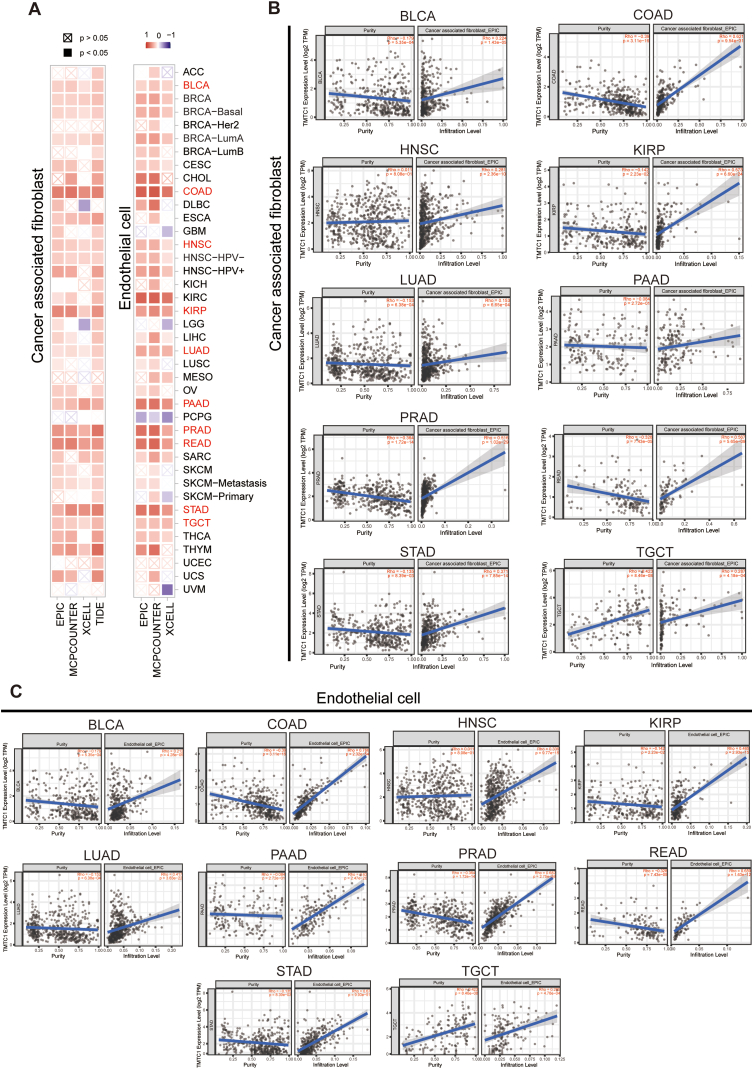
Immune infiltration analysis of *TMTC1*. (A) The heatmap showed the correlation between *TMTC1* expression and infiltration level of cancer associated fibroblast and endothelial cell. **(B**–**C)** The scatter plots showed Spearman's correlation between *TMTC1* expression and infiltration level of cancer associated fibroblast **(B)** and endothelial cell **(C)** in BLCA, COAD, HNSC, KIRP, LUAD, PAAD, PRAD, READ, STAD, and TGCT (EPIC algorithm).

To investigate the relationship between *TMTC1* expression and immune infiltration, we utilized SangerBox3.0 to compute immune infiltration scores for 33 types of cancer. Subsequently, we examined the Pearson's correlation between *TMTC1* expression and the stromal score, immune score, and ESTIMATE score for these 33 cancers. As shown in Fig. 4A, a significant positive correlation was observed between *TMTC1* expression and stromal score across 21 cancers. Scatter plots in Fig. 4B further illustrated this strong correlation between *TMTC1* expression and stromal score in 12 specific cancers (BLCA, BRCA, COAD, KIRC, KIRP, LAML, PAAD, PRAD, READ, SARC, STAD, and THCA). Given the significant correlation between *TMTC1* expression and stromal score in most cancers, we further evaluated the relationship between *TMTC1* expression and infiltration levels of stromal cells, such as cancer-associated fibroblasts (CAFs) and endothelial cells, which were important components of the tumor microenvironment and contributed to tumor development. As shown in Fig. 5A, a significant positive correlation was observed between *TMTC1* expression and the infiltration levels of these two cell types in most cancers through different algorithms (EPIC, MCP-COUNTER, XCELL, and TIDE). Scatter plots in Fig. 5B and C demonstrate significant correlations between *TMTC1* expression and infiltration levels of CAFs and endothelial cells, respectively, in 10 specific cancer types (BLCA, COAD, HNSC, KIRP, LUAD, PAAD, PRAD, READ, STAD, and TGCT) through EPIC algorithm.

### 3.5 Single-cell analysis of TMTC1

**Fig. 6:**
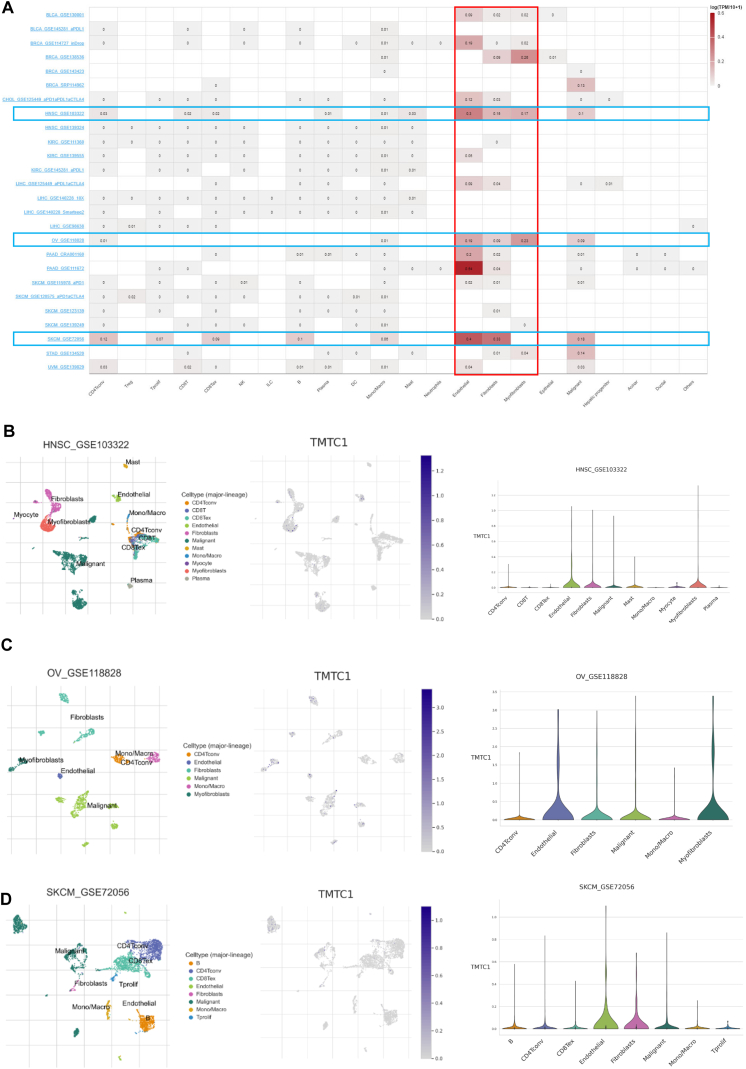
The mRNA expression levels of *TMTC1* in all cells in tumor microenvironment. (A) Summary of *TMTC1* expression of 22 cell types in 26 single cell databases. **(B**–**D)** Scatter plots was used to display the distributions of various cell types (left) and their respective *TMTC1* expression levels (middle), while a violin plot (right) was employed to illustrate the *TMTC1* expression across different cell populations in the GSE103322 HNSC database **(B)**, GSE118828 OV database **(C)**, and GSE72056 SKCM database **(D)**.

The tumor microenvironment consists of a heterogeneous collection of cell types, including immune cells, endothelial cells, fibroblasts, and cancer cells. To investigate the expression of *TMTC1* in different cell types within the tumor microenvironment, we utilized the TISCH database. As shown in Fig. 6A, *TMTC1* demonstrates higher expression levels in stromal cells, particularly fibroblasts, endothelial cells, and myofibroblasts, compared to immune cells in most cancers. Furthermore, the scatter plots in Fig. 6B–D also illustrated that *TMTC1* is highly expressed in fibroblasts, endothelia cells and myofibroblasts in the tumor microenvironment in HNSC, OV, and SKCM.

### 3.6 Immunotherapy analysis of TMTC1

**Fig. 7:**
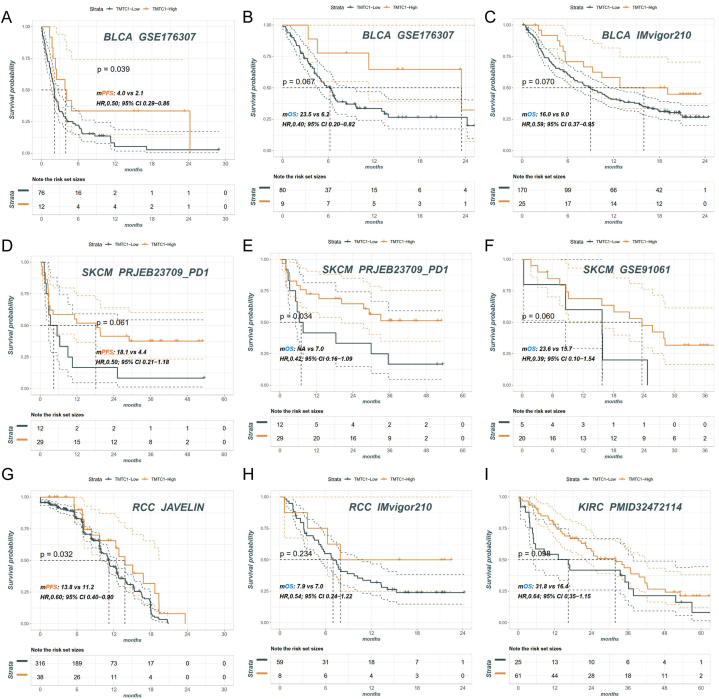
Correlation of *TMTC1* expression with immunotherapy response. Kaplan–Meier curve of survival analysis of *TMTC1* in BLCA **(A**–**C)**, SKCM **(D**–**F)**, RCC **(G**–**H)** and KIRC **(I)** immunotherapy cohort.

To investigate the correlation between the expression level of *TMTC1* and the efficacy of immunotherapy in tumors, we conducted a comparative analysis of prognostic outcomes among cancer patients with high and low *TMTC1* expression following treatment with immune checkpoint inhibitors. Our analysis included data from various cohorts: the BLCA cohort from GSE176307 (Fig. 7A and B) and IMvigor210 (Fig. 7C), the SKCM cohort from PRJEB23709 (Fig. 7D and E) and GSE91061 (Fig. 7F), the RCC cohort from JAVELIN (Fig. 7G) and IMvigor210 (Fig. 7H), and the KIRC cohort from PMID32472114 (Fig. 7I). The results consistently indicated that patients with high *TMTC1* expression had a longer PFS or OS compared to those with low *TMTC1* expression.

### 3.7 Drug sensitivity analysis of TMTC1

**Fig. 8:**
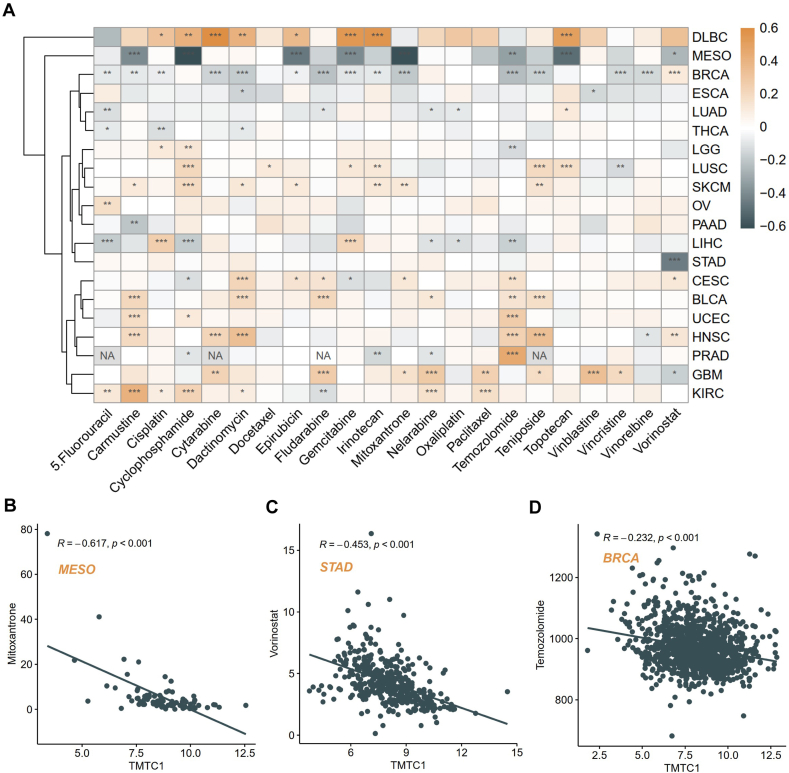
Drug sensitivity analysis of *TMTC1*. (A) Correlation of *TMTC1* expression with the sensitivity of 22 common drugs in pan-cancer. **(B**–**D)** The scatter plot of the top negatively correlated drugs and *TMTC1*, such as mitoxantrone in MESO **(B)**, vorinostat in STAD **(C)**, and temozolomide in BRCA **(D)**.

To examine the potential influence of *TMTC1* on patients’ response to chemotherapy, we downloaded the IC50 (half-maximal inhibitory concentration) and expression data in cancer cell lines from the Genomics of Drug Sensitivity in Cancer (GDSC) database and then utilized the 'OncoPredict' package to predict the drug sensitivity of patients from TCGA across 20 cancer types. Subsequently, we examined the correlation between *TMTC1* expression levels and the predicted drug sensitivity. As shown in Fig. 8A, our analysis revealed significant positive associations between *TMTC1* expression and the sensitivity of 22 common antitumor drugs in multiple tumor types, including DLBC, SKCM, BLCA, and GBM. Conversely, a marked negative correlation was observed between *TMTC1* expression and the sensitivity of these drugs in MESO and RBCA. Specifically, the overexpression of *TMTC1* significantly decreased the IC50 values of mitoxantrone in MESO (Fig. 8B), vorinostat in STAD (Fig. 8C), and temozolomide in BRCA (Fig. 8D). This suggests that elevated *TMTC1* expression reduces the resistance of tumor cells to these drugs, thereby enhancing their effectiveness in treatment.

### 3.8 TMTC1-related gene enrichment analysis

**Fig. 9:**
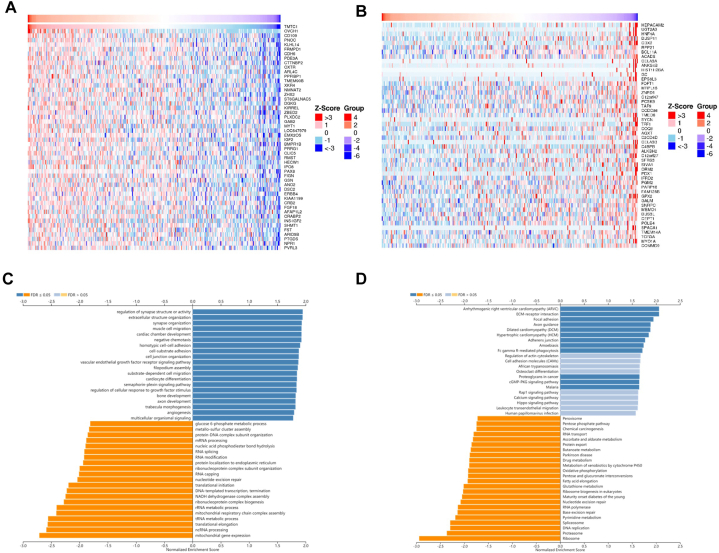
The analyses of *TMTC1*-related genes in ovarian serous cystadenocarcinoma. Heatmap of the top 50 positive **(A)** and negative **(B)** associated genes of *TMTC1* in ovarian serous cystadenocarcinoma. Bar plot of GO **(C)** and KEGG **(D)** enrichment analysis for 100 TMTC-related genes (50 positive associated genes and 50 negative associated gene) using GSEA in the ovarian serous cystadenocarcinoma cohort.

To investigate the functions of *TMTC1* in cancer, we utilized the LinkedOmics tool to identify 100 genes that were related with *TMTC1* in ovarian serous cystadenocarcinoma. Fig.s 9A and 9B present heat maps of 50 genes that are significantly positively correlated and 50 genes that are significantly negatively correlated with *TMTC1* expression, respectively. Through Gene Set Enrichment Analysis, which was conducted using the 100 *TMTC1*-related genes in ovarian serous cystadenocarcinoma, it was found that GO biological process terms primarily showed enrichment in regulation of synapse structure or activity, extracellular structure organization, synapse organization, muscle cell migration, cardiac chamber development, negative chemotaxis, homotypic cell-cell adhesion, cell-substrate adhesion, cell junction organization, vascular endothelial growth factor receptor signaling pathway, filopodium assembly, substrate-dependent cell migration, cardiocyte differentiation, semaphorin-plexin signaling pathway, regulation of cellular response to growth factor stimulus, bone development, axon development, trabecula morphogenesis, and angiogenesis (Fig. 9C). Kyoto Encyclopedia of Genes and Genomes (KEGG) pathway enrichment analysis showed that the major enriched pathways were arrhythmogenic right ventricular cardiomyopathy (ARVC), ECM-receptor interaction, focal adhesion, axon guidance, dilated cardiomyopathy (DCM), hypertrophic cardiomyopathy (HCM), adherens junction, amoebiasis, Fc gamma R-mediated phagocytosis, proteoglycans in cancer, and GMP-PKG signaling pathway (Fig. 9D).

## 4 Discussion

*TMTC1* is an essential ER protein that helps maintain ER calcium balance [5] and is involved in protein glycosylation, crucial for protein folding, stability, and function [7,9,43]. Dysregulation of *TMTC1* leads to altered intracellular Ca2+ levels and impaired glycosylation, both linked to cancer development and progression [[44], [45], [46], [47]]. Although *TMTC1* dysregulation is associated with several cancers. [10,12,16,17], a comprehensive pan-cancer analysis was lacking. Our study addresses this by examining *TMTC1*'s expression, prognosis, genetic alterations, immune infiltration, immunotherapy response, drug sensitivity, and regulatory mechanisms across various cancers, aiming to explore the prognostic significance of *TMTC1* and its potential as an immunotherapeutic target.

Firstly, our analysis revealed that *TMTC1* exhibits lower expression in 19 cancers (ACC, BLCA, BRCA, CESC, COAD, ESCA, GBM, KICH, KIRC, KIRP, LAML, LUAD, LUSC, PRAD, READ, STAD, THCA, UCEC, and UCS) compared to normal tissues. This finding was consistent with previous research on *TMTC1* in GBM [17], BRCA [15], and STAD [14]. The downregulation of *TMTC1* in cancer may have significant implications for tumor progression., as reduced *TMTC1* expression may lead to a decrease in O-mannosylation of the tumor suppressor protein E-cadherin, which promoted tumor spread [7,46]. Additionally, our analysis demonstrated that elevated expression of *TMTC1* is associated with poor prognosis in several cancers, including ACC, BLCA, COAD, GBM, KIRP, OV, STAD, and UCEC. These findings were consistent with previous studies conducted by Yeh et al. in ovarian cancer [8], Xin Chen et al. in gastric cancer [14], and McInerney et al. in GBM [17]. Conversely, *TMTC1* expression showed a protective effect on survival in patients with CESC, KIRC, LUSC, and PAAD. These findings highlight the heterogeneity of *TMTC1*'s prognostic significance across different cancer types and emphasize its potential as a biomarker for predicting patient outcomes.

Mutations or functional loss in TMTC proteins are closely associated with various diseases, such as hearing loss [[48], [49], [50]], neuronal cell migration disorders [51,52], brain development abnormalities [51], and brain malformations [52]. Our study is the first to analyze the relationship between *TMTC1* genetic alterations and cancer. Our findings show that *TMTC1* alterations, primarily mutations and amplifications, are most frequent in LUAD, OV, and TGCT. These genetic alterations of *TMTC1* might be related to the mechanisms and molecular characteristics underlying different tumor types. Additionally, *TMTC1* genetic alterations significantly affect patient survival across various cancers, suggesting *TMTC1* mutations could be potential prognostic markers for survival and treatment response.

Our analysis found a significant positive correlation between *TMTC1* expression and stromal score across various cancers, suggesting *TMTC1*'s role in stromal cell infiltration. *TMTC1* expression also correlated positively with cancer-associated fibroblasts (CAFs) and endothelial cell infiltration. CAFs promote tumor cell proliferation, migration, and invasion by secreting growth factors, cytokines and extracellular matrix proteins [53]. Endothelial cells support tumor angiogenesis by producing factors like VEGF and PDGF. The association between *TMTC1* expression and the infiltration of CAFs and endothelial cells emphasizes its potential involvement in cancer metastasis. Single-cell analysis using the TISCH database showed higher *TMTC1* expression in stromal cells, particularly fibroblasts, endothelial cells, and myofibroblasts, compared to immune cells. This suggests *TMTC1* plays a significant role in the non-immune components of the tumor microenvironment.

Immunotherapy has emerged as a promising treatment approach for various cancers [54]. One of the key strategies in immunotherapy is the use of immune checkpoint inhibitors, which are drugs that block the inhibitory signals that allow cancer cells to evade the immune system [55]. Our study reveals that high *TMTC1* expression is associated with improved prognosis in cancer patients receiving immune checkpoint inhibitors. Specifically, patients with elevated *TMTC1* levels show longer progression-free survival (PFS) and overall survival (OS) compared to those with low *TMTC1* expression. These results suggest that *TMTC1* could be an important factor in predicting and enhancing the effectiveness of immunotherapy.

Chemotherapy is a common treatment approach for cancer, which utilizes drugs to destroy or inhibit the growth of cancer cells in the body [[56], [57], [58]]. Our analysis explored the potential influence of *TMTC1* on patients' response to chemotherapy. We observed significant associations between *TMTC1* expression and the sensitivity of several common antitumor drugs in different tumor types. This analysis highlights the potential role of *TMTC1* in mediating drug sensitivity in various cancer types. It suggests that targeting *TMTC1* and modulating its expression could be a promising strategy to improve the efficacy of chemotherapy in certain tumor types. Further studies are warranted to elucidate the underlying mechanisms and validate these findings in clinical settings.

Recent research has revealed that *TMTC1* promotes the occurrence and progression of ovarian cancer, indicating its potential as a therapeutic target [8]. To further investigate the molecular mechanisms of *TMTC1* in ovarian cancer, we conducted gene enrichment analysis using *TMTC1*-related genes in ovarian serous cystadenocarcinoma. The results revealed significant enrichment in biological processes and pathways associated with cell migration, adhesion, and angiogenesis. Cell migration played a crucial role in the development and progression of cancer by facilitating the movement of cancer cells from the primary tumor site to distant organs, thereby promoting metastasis [59]. Cell adhesion refers to the ability of cancer cells to attach to neighboring cells or the surrounding extracellular matrix. Alterations in the adhesion properties of cancer cells endow them with an invasive and migratory phenotype [60]. Moreover, angiogenesis played a critical role in tumor growth by promoting the formation of new blood vessels to provide nutrients and oxygen to the growing tumor [61]. The significant correlation between *TMTC1* and these processes suggested that *TMTC1* may contribute to cancer aggressiveness and metastasis by directly or indirectly influencing cell migration, adhesion, and angiogenesis.

Our study is the reliance on publicly available datasets, which may introduce biases and inconsistencies in data collection and processing. Additionally, the regulatory mechanisms underlying the dysregulation of *TMTC1* in cancer remain largely unknown. Therefore, future studies should focus on elucidating these mechanisms and exploring potential therapeutic strategies targeting *TMTC1*. Furthermore, the functional role of *TMTC1* in cancer was primarily explored through bioinformatics analysis, and further experimental validation is necessary to confirm these findings.

## 5 Conclusion

In conclusion, this study comprehensively analyzed the expression, prognosis, genetic alterations, immune infiltration, immunotherapy response, drug sensitivity, and regulatory mechanisms of *TMTC1* in pan-cancer. The findings highlight the prognostic significance of *TMTC1* and its potential as an immunotherapeutic target.

## Data availability statement

The RNA sequencing and paired OS outcomes of thirty-three cancer types form TCGA were acquired from the UCSC Xena database (https://xenabrowser.net/datapages/). The PD1/PD-L1 therapy cohorts (IMvigor210 cohort and GSE176307) from the GEO database and studies conducted by Balar [62]. The IC50 values and transcriptomic data for cell lines treated with 198 drugs were downloaded from the GDSC2 database (https://www.cancerrxgene.org). Readers could download all data included in this study by searching accession numbers mentioned in Data acquisition of Method.

## Funding

This work did not receive any specific grant from funding agencies in the public, commercial, or not-for-profit sectors.

## Ethics statement

All the data included in the analysis were obtained from public databases without the need of permissions from local ethical committees.

## CRediT authorship contribution statement

**Ying Zhang:** Writing – review & editing, Writing – original draft. **Dan Wu:** Writing – review & editing, Writing – original draft. **Tiantian Yu:** Formal analysis, Data curation. **Yao Liu:** Methodology, Formal analysis, Data curation. **Chunbo Zhao:** Writing – review & editing, Visualization, Conceptualization. **Ruihong Xue:** Conceptualization.

## Declaration of competing interest

The authors declare the following financial interests/personal relationships which may be considered as potential competing interests:There no any other relationship or activity that may be interpreted as a conflict of interest by the reader. If there are other authors, they declare that they have no known competing financial interests or personal relationships that could have appeared to influence the work reported in this paper.
